# Emognition dataset: emotion recognition with self-reports, facial expressions, and physiology using wearables

**DOI:** 10.1038/s41597-022-01262-0

**Published:** 2022-04-07

**Authors:** Stanisław Saganowski, Joanna Komoszyńska, Maciej Behnke, Bartosz Perz, Dominika Kunc, Bartłomiej Klich, Łukasz D. Kaczmarek, Przemysław Kazienko

**Affiliations:** 1 Wroclaw University of Science and Technology, Faculty of Information and Communication Technology, Department of Artificial Intelligence, Wrocław, 50-370 Poland; 2 Adam Mickiewicz University, Faculty of Psychology and Cognitive Science, Poznan, 61-664 Poland

**Keywords:** Psychology, Computer science

## Abstract

The Emognition dataset is dedicated to testing methods for emotion recognition (ER) from physiological responses and facial expressions. We collected data from 43 participants who watched short film clips eliciting nine discrete emotions: amusement, awe, enthusiasm, liking, surprise, anger, disgust, fear, and sadness. Three wearables were used to record physiological data: EEG, BVP (2x), HR, EDA, SKT, ACC (3x), and GYRO (2x); in parallel with the upper-body videos. After each film clip, participants completed two types of self-reports: (1) related to nine discrete emotions and (2) three affective dimensions: valence, arousal, and motivation. The obtained data facilitates various ER approaches, e.g., multimodal ER, EEG- vs. cardiovascular-based ER, discrete to dimensional representation transitions. The technical validation indicated that watching film clips elicited the targeted emotions. It also supported signals’ high quality.

## Abstract (summary)

**Table:** 

Measurement(s)	cardiac output measurement • Electroencephalography • Galvanic Skin Response • Temperature • acceleration • facial expressions
Technology Type(s)	photoplethysmogram • electroencephalogram (5 electrodes) • electrodermal activity measurement • Sensor • Accelerometer • Video Recording
Sample Characteristic - Organism	Homo sapiens
Sample Characteristic - Environment	laboratory environment

## Background & Summary

The ability to recognize human emotions based on physiology and facial expressions opens up important research and application opportunities, mainly in healthcare and human-computer interaction. Continuous affect assessment can help patients suffering from affective disorders^1^ and children with autism spectrum disorder^2^. On a larger scale, promoting emotional well-being is likely to increase public health, improve the quality of life, and prevent some mental problems^3,4^. Emotion recognition could also enhance interaction with robots – they would better and less obtrusively understand the user’s commands, needs, and preferences^5^. Furthermore, difficulty in video games could be adjusted to the user’s emotional feedback^6^. Recommendations for movies, music, search engine results, user interface, and content may be enriched with the user’s emotional context^7,8^.

To achieve market-ready and evidence-based solutions, the machine learning models detecting and classifying affect and emotions need improvement. Such models require a large amount of data collected from several affective outputs to train complex, data-intensive deep learning architectures. Over the last decade, several datasets on physiological responses and facial expressions to affective stimuli have been published, i.e., POPANE^9^, BIRAFFE^10^, QAMAF^11^, ASCERTAIN^12^, DECAF^13^, MAHNOB-HCI^14^, and DEAP^15^. However, these datasets have limitations such as using only dimensional scales to capture participants’ emotional state rather than asking about discrete emotions.

The Emognition dataset contains physiological signals and upper-body recordings of 43 participants who watched validated emotionally arousing film clips targeted at nine discrete emotions. The autonomous nervous system responses to the stimuli were recorded with consumer-grade wearables: Muse 2 equipped with electroencephalograph (EEG), accelerometer (ACC), and gyroscope (GYRO) sensors; Empatica E4 measuring and providing blood volume pulse (BVP), electrodermal activity (EDA), skin temperature (SKT), and also providing interbeat interval (IBI), and ACC data; Samsung Galaxy Watch measuring and providing BVP, and also providing heart rate (HR), peak-to-peak interval (PPI), ACC, GYRO, and rotation data. The participants reported their emotions using discrete and dimensional questionnaires. The technical validation supported that participants experienced targeted emotions and the obtained signals are of high quality.

The Emognition dataset offers the following advantages over the previous datasets: (1) the physiological signals have been recorded using wearables which can be applied unobtrusively in everyday life scenarios; (2) the emotional state has been represented with two types of emotional models, i.e., discrete and dimensional; (3) nine distinct emotions were reported; (4) we put an emphasis on the differentiation between positive emotions; thus, this is the only dataset featuring four discrete positive emotions; the differentiation is important because studies indicated that specific positive emotions might differ in their physiology^16–18^; (5) the dataset enables versatile analyses within emotion recognition (ER) from physiology and facial expressions.

The Emognition dataset may serve to tackle the research questions related to: (1) multimodal approach to ER; (2) physiology-based ER vs. ER from facial expressions; (3) ER from EEG vs. ER from BVP; (4) ER with Empatica E4 vs. ER using Samsung Watch (both providing BVP signal collected in parallel); (5) classification of positive vs. negative emotions; (6) affect recognition – low vs. high arousal and valence; (7) analyses between discrete and dimensional models of emotions.

## Methods

### Ethics statement

The study was approved by and performed in accordance with the guidelines and regulations of the Wroclaw Medical University, Poland; approval no. 149/2020. The submission to the Ethical Committee covered, among others, participant consent, research plans, recruitment strategy, data management procedures, and GDPR issues. Participants provided written informed consent, in which they declared that they (1) were informed about the study details, (2) understand what the research involves, (3) understand what their consent was needed for; (4) may refuse to participate in the research at any time during the research project; (5) had the opportunity to ask questions of the experimenter and receive answers to those questions. Finally, participants gave informed consent to participate in the research, agreed to be recorded during the study, and consented to the processing of their personal data to the extent necessary for the implementation of the research project, including sharing their psycho-physiological and behavioral data with other researchers.

### Participants

The participants were recruited via a paid advertisement on Facebook. Seventy people responded to the advertisement. We have excluded ten non-Polish speaking volunteers. An additional 15 could not find a suitable date, and two did not show up for the scheduled study. As a result, we collected data from 43 participants (21 females) aged between 19 and 29 (*M* = 22.37, *SD* = 2.25). All participants were Polish.

The exclusion criteria were significant health problems, use of drugs and medications that might affect cardiovascular function, prior diagnosis of cardiovascular disease, hypertension, or BMI over 30 (classified as obesity). We asked participants to reschedule if they experienced an illness or a major negative life event. The participants were requested (1) not to drink alcohol and not to take psychoactive drugs 24 hours before the study; (2) to refrain from caffeine, smoking, and taking nonprescription medications for two hours before the study; (3) to avoid vigorous exercise and eating an hour before the study. Such measures were undertaken to eliminate factors that could affect cardiovascular function.

All participants provided written informed consent and received a 50 PLN (c.a., $15) online store voucher.

### Stimuli

We used short film clips from databases with prior evidence of reliability and validity in eliciting targeted emotions^19–23^. The source film, selected scene, and stimulus duration are provided in Table 1.

**Table 1:** Stimuli clips used to elicit emotions.

Targeted emotion (Polish translation)	Source film	Scene	Duration [min.]	Ref.
Anger (złość)	American History X	A neo-nazi smashes a Black man’s head on the curb killing him	02:00	^22^
Fear (strach)	The Blair Witch Project	The clip begins with suspense and ends with an intense burst	02:00	^22^
Surprise (zaskoczenie)	Capricorn One	Unexpectedly, men are bursting through the door	00:49	^21^
Sadness (smutek)	Champ	A boy cries at the death of his father	01:59	^21^
Disgust (obrzydzenie)	Trainspotting 2	A man suffering from violent diarrhea goes to an extremely dirty public restroom	01:08	^22^
Amusement (rozbawienie)	A Fish Called Wanda	Unexpectedly, the owners of the house get into the house and discover Archie dancing naked	02:00	^21^
Enthusiasm (entuzjazm)	London 2012	A montage of moments showing athletes’ successful performance and their joyful reactions	01:59	^20^
Awe (zachwyt)	NEW York from PONE	A montage of architecture in a modern city	01:56	^20^
Liking (pragnienie)	Food	A presentation of desserts	01:51	^20^
Neutral (neutralny)	Blue	A woman goes up an escalator, carrying a box	02:01	^22^

### Measures

**Fig. 1:**
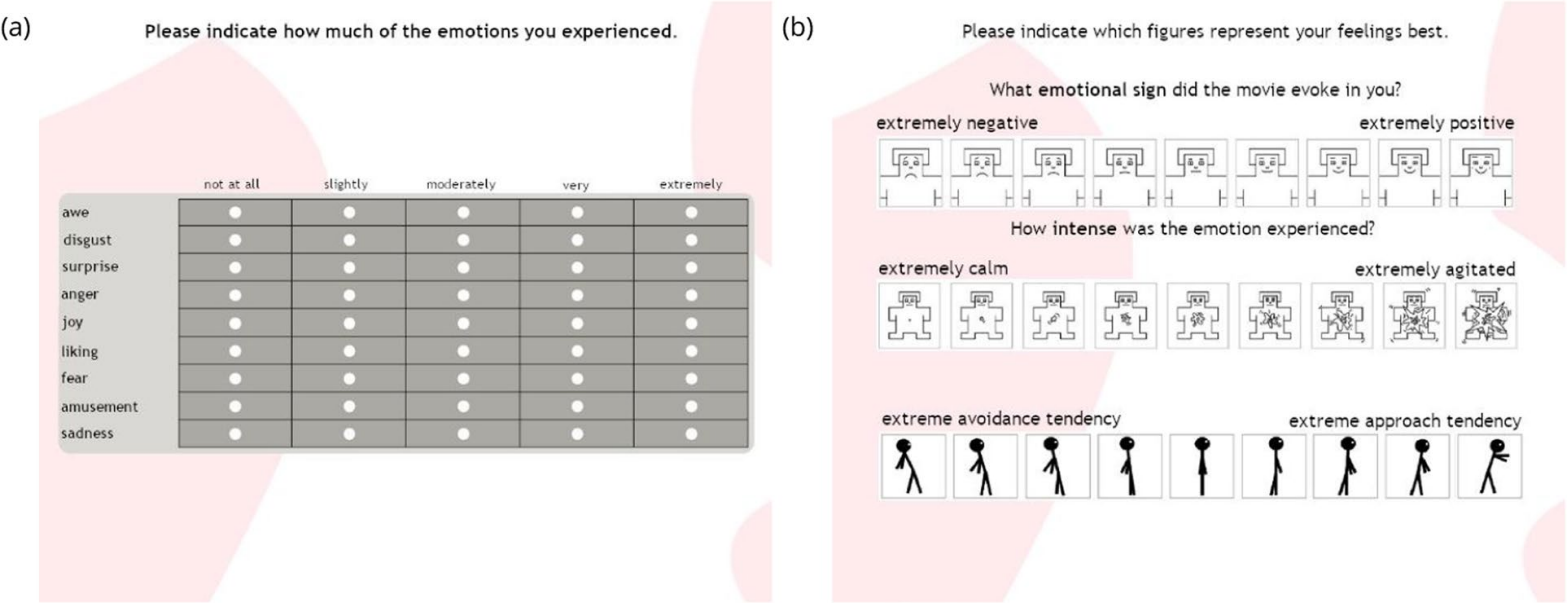
The English version of the self-reports used in the study: (a) questionnaire for discrete emotions; (b) questionnaire for valence, arousal, and motivation. The original Polish version can be found in the Supp. Mat. Fig 3.

We used two types of self-assessment for manipulation checks that accounted for discrete and dimensional approaches to emotions. For the discrete approach, participants reported retrospectively, using single-item rating scales, on how much of the targeted emotions they had experienced while watching the film clips^21^. The questionnaire was filled in electronically with a tablet, see Fig. 1a. It included nine items corresponding to the selected stimuli. Each emotion-related scale ranged from 1 (not at all) to 5 (extremely). The questionnaire was modeled after the instruments used in previous studies with similar methodology^24–27^.

For the dimensional approach, participants reported retrospectively, using single-item rating scales, on how much valence, arousal, and motivation they experienced while watching the film clips. The 3-dimensional emotional self-report was collected with the Self-Assessment Manikin – SAM^28^. The SAM is a validated nonverbal visual assessment developed to measure affective responses. Participants reported felt emotions using a graphical scale ranging from 1 (a very sad figure) to 9 (a very happy figure) for valence, Fig. 1b; and from 1 (a calm figure) to 9 (an agitated figure) for arousal, Fig. 1b. We also asked participants to report their motivational tendency using a validated graphical scale modeled after the SAM^29^, i.e., whether they felt the urge to avoid or approach while watching the film clips, from 1 (figure leaning backward) to 9 (figure leaning forward)^30^, Fig. 1b. The English versions of the self-reports used in the study are illustrated in Fig. 1.

### Apparatus

The behavioral and physiological signals were gathered using three wearable devices and a smartphone:

- An **EEG headband Muse 2** equipped with four EEG electrodes (AF7, AF8, TP9, and TP10), accelerometer (ACC), and gyroscope (GYRO). The data was transmitted to a smartphone in real-time using the *Mind Monitor* (https://mind-monitor.com) application. At the end of each day, data from the smartphone was transferred to the secure disk;
- A **wristband Empatica E4** monitoring blood volume pulse (BVP), interbeat interval (IBI), electrodermal activity (EDA), acceleration, and skin temperature (SKT). The Empatica E4 was mounted on the participant’s dominant hand. The device was connected wirelessly via Bluetooth to the tablet using a custom-made Android application with Empatica E4 link SDK module^31^. The data was streamed in real-time to the tablet and after the study to the secure server. The signals obtained with the Empatica E4 were synchronized with the stimuli presented on the tablet;
- A **smartwatch Samsung Galaxy Watch SM-R810** providing heart rate (HR), peak-to-peak interval (PPI), raw BVP – the amount of reflected LED light, ACC, GYRO, and rotation data. A custom Tizen application was developed and installed on the watch to collect and store data locally. At the end of each day, data was downloaded to the secure disk;
- A **smartphone Samsung Galaxy S20** + **5** **G** recording participants’ upper-body – head, chest, and hands. The footage also included a small mirror reflecting the tablet screen to enable later synchronization with stimuli. At the end of each day, recordings were moved to the encrypted offline disk.

The Muse 2 has lower reliability than medical devices but sufficient for nonclinical trial settings^32^. It has been successfully used to observe and quantify event-related brain potentials^33^, as well as to recognize emotions^34^. The Empatica E4 has been compared with a medical electrocardiograph (ECG), and proved to be a practical and valid tool for studies on HR and heart rate variability (HRV) in stationary conditions^35^. It was also likewise effective as the Biopac MP150 in the emotion recognition task^36^. Moreover, we have used the Empatica E4 for intense emotion detection with promising results in a field study^37,38^. The Samsung Watch devices were successfully utilized (1) to track the atrial fibrillation with an ECG patch as a reference^39^, and (2) to assess the sleep quality with a medically approved actigraphy device as a baseline^40^. Moreover, Samsung Watch 3 performed well in detecting intense emotions^41^.

Additionally, a 10.4-inch tablet Samsung Galaxy Tab S6 was used to guide participants through the study. A dedicated application was developed to instruct the participants, present stimuli, collect self-assessments, as well as gather Empatica E4 signals, and synchronize them with the stimuli.

**Fig. 2:**
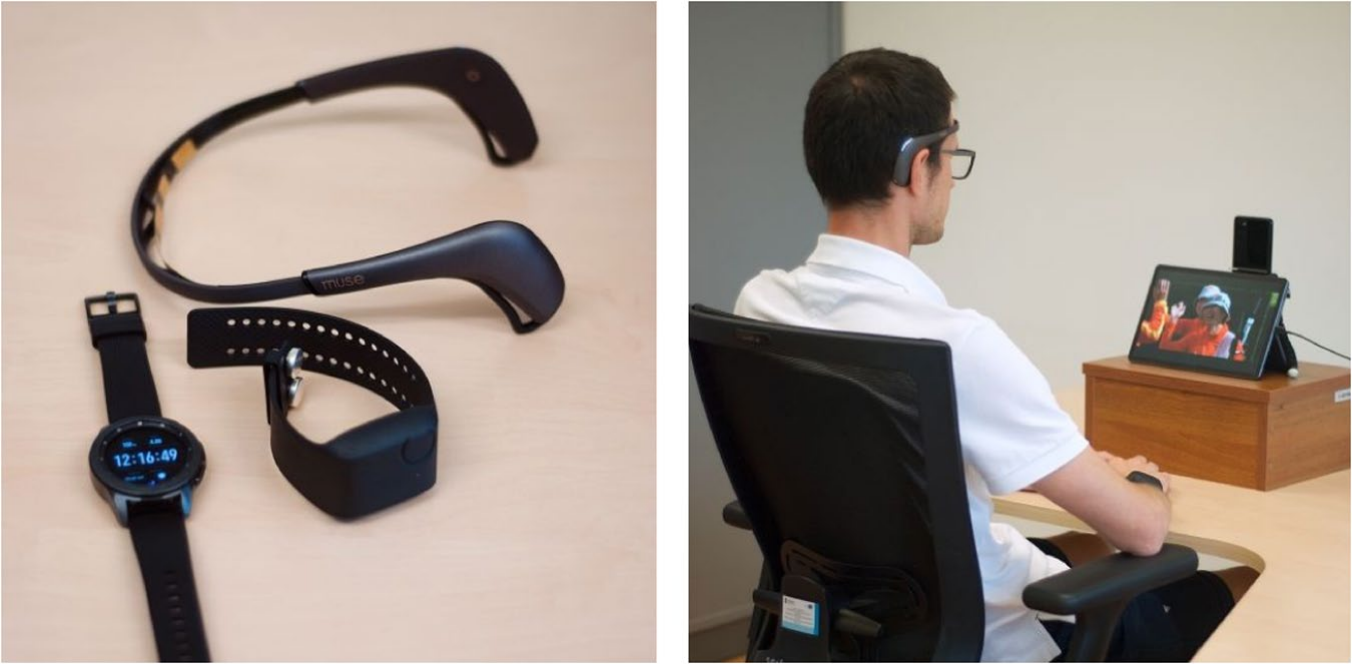
Devices used to gather the physiological data and the experimental stand.

The sampling rate of the collected signals is provided in Table 2. The devices and the experimental stand are illustrated in Fig. 2.

**Table 2:** Sampling rate of signals and other data available in the Emognition dataset.

Device	Signal/Data	Sampling rate
Empatica E4	Blood volume pulse	64 Hz
	Interbeat interval	Variable
	Electrodermal activity	4 Hz
	3-axis accelerometer	32 Hz
	Skin temperature	4 Hz
Samsung Galaxy Watch	Heart rate	10 Hz
	Peak-to-peak interval	10 Hz
	Raw blood volume pulse	20 Hz
	Processed blood volume pulse	20 Hz
	3-axis accelerometer	33 Hz
	3-axis gyroscope	33 Hz
	4-axis rotation	33 Hz
Muse 2	Data from AF7, AF8, AF9, and AF10 electrodes: Raw EEG signal; absolute band powers for Alpha, Beta, Gamma, Delta, Theta	256 Hz
	3-axis accelerometer	256 Hz
	3-axis gyroscope	256 Hz
Samsung Galaxy S20 + 5 G	Upper-body video recording	60 fps

### Procedure

**Fig. 3:**
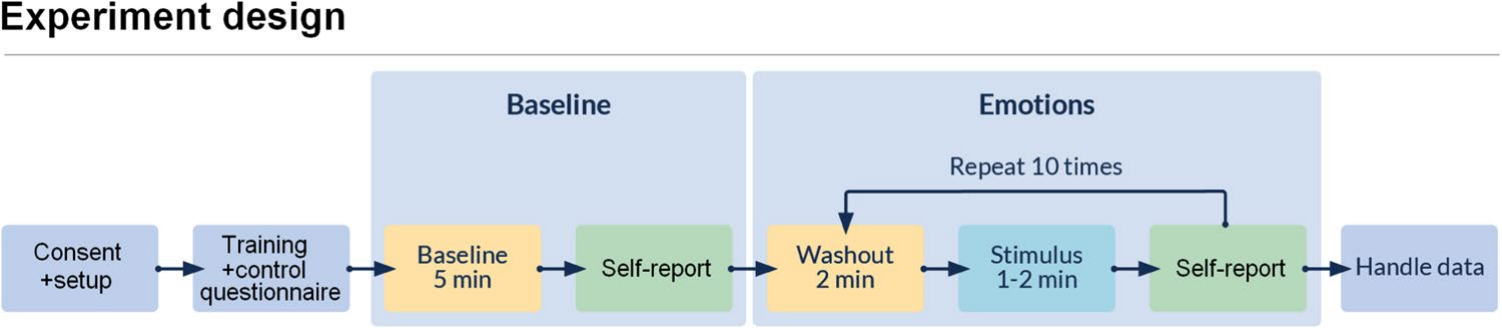
The experiment procedure.

The study was conducted between the 16th of July and the 4th of August, 2020. It took place in the UX Wro Lab - a laboratory at the Wrocław University of Science and Technology. Upon arrival, participants were informed about the experimental procedure, Fig. 3. They then signed the written consent. The researcher applied the devices approximately five minutes before the experiment so that the participants could get familiar with them. It also enabled a proper skin temperature measurement. From this stage until the end of the experiment, the physiological signals were recorded. Next, participants listened to instructions about the control questionnaire and self-assessments. The participants filled out the control questionnaire about their activity before the experiment, e.g., time since the last meal or physical activity and wake-up time. Their responses are part of the dataset.

The participants were asked to avoid unnecessary actions or movements (e.g., swinging on the chair) and not to cover their faces. They were also informed that they could skip any film clip or quit the experiment at any moment. Once the procedure was clear to the participants, they were left alone in the room but could ask the researcher for help anytime. For the baseline, participants watched dots and lines on a black screen for 5 minutes (physiological baseline) and reported current emotions (emotional baseline) using discrete and dimensional measures. The main part of the experiment consisted of ten iterations of (1) a 2-minute washout clip (dots and lines), (2) the emotional film clip, and (3) two self-assessments, see Fig. 3. The order of film clips was counterbalanced using a Latin square, i.e., we randomized clips for the first participant and then shifted by one film clip for each next participant so that the first film clip was placed as the last one.

After the experiment, participants provided information about which movies they had seen before the study and other remarks about the experiment. Concluding the procedure, participants received the voucher. The whole experiment lasted about 50 minutes, depending on the time spent on the questionnaires.

### Data processing and cleaning

**Fig. 4:**
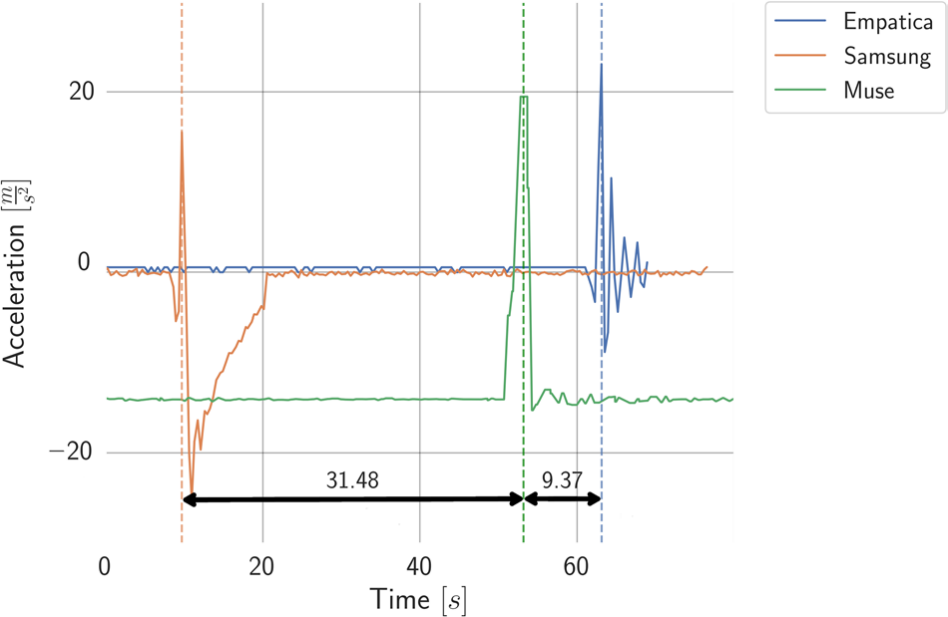
The time difference between the devices used in the study identified by recording the ACC signal when devices were moved according to the synchronization procedure.

Empatica E4 was synchronized with the stimuli *out-of-the-box* using a custom application and Empatica E4 SDK. Samsung Watch and Muse 2 devices were synchronized using accelerometer signals. All three devices were placed on the table, which was then hit with a fist. The first peak in the ACC signal was used to find the time shift between the devices, Fig. 4. All times were synchronized to the Empatica E4 time.

Each device stored data in a different format and structure. We unified the data to JSON format and divided the experiment into segments covering washouts, film clips, and self-assessment separately. We provide the raw recordings from all used devices. Additionally, we performed further preprocessing for some devices/data and provide it alongside the raw data.

For EEG, the *raw signal* represents the signal filtered with a 50 Hz notch frequency filter, which is a standard procedure to remove interference caused by power lines. Besides the raw EEG, the *Mind Monitor* application provides the absolute band power for each channel and five standard frequency ranges (i.e., delta to gamma, see Table 2). According to the *Mind Monitor* documentation, these are obtained by (1) using a fast Fourier transform (FFT) to compute the power spectral density (PSD) for frequencies in each channel, (2) summing the PSDs over a frequency range, and (3) taking the logarithm of the sum, to get the result in Bels (B). The *Mind Monitor* documentation presents details https://mind-monitor.com.

The processing of BVP signal from the Samsung Watch PPG sensor consisted of subtracting the mean component, eight-level decomposition using Coiflet1 wavelet transform, and then reconstructing it by the inverse wavelet transform based only on the second and third levels. Amplitude fluctuations were reduced by dividing the middle value of the signal by the standard deviation of a one second long sliding window with an odd number of samples. The final step was signal normalization to the range of [−1,1].

The upper-body recordings were processed with the *OpenFace* toolkit^42–44^ (version 2.2.0, default parameters) and *Quantum Sense* software (Research Edition 2017, Quantum CX, Poland). The *OpenFace* library provides facial landmark points and action units’ values, whereas *Quantum Sense* recognizes basic emotions (neutral, anger, disgust, happiness, sadness, surprise) and head pose.

Some parts of the signals were of lower quality due to the participants’ movement or improper mounting. For example, the quality of EEG signal can be investigated using Horse Shoe Indicator (HSI) values provided by the device, which represent how well the electrodes fit the participant’s head. For video clips, *OpenFace* provides information about detected faces with their head pose per one frame. We have not removed low-quality signals so that users of the dataset can decide how to deal with them. Any data-related problems that we identified are included in the *data_completeness.csv* file.

## Data Records

**Fig. 5:**
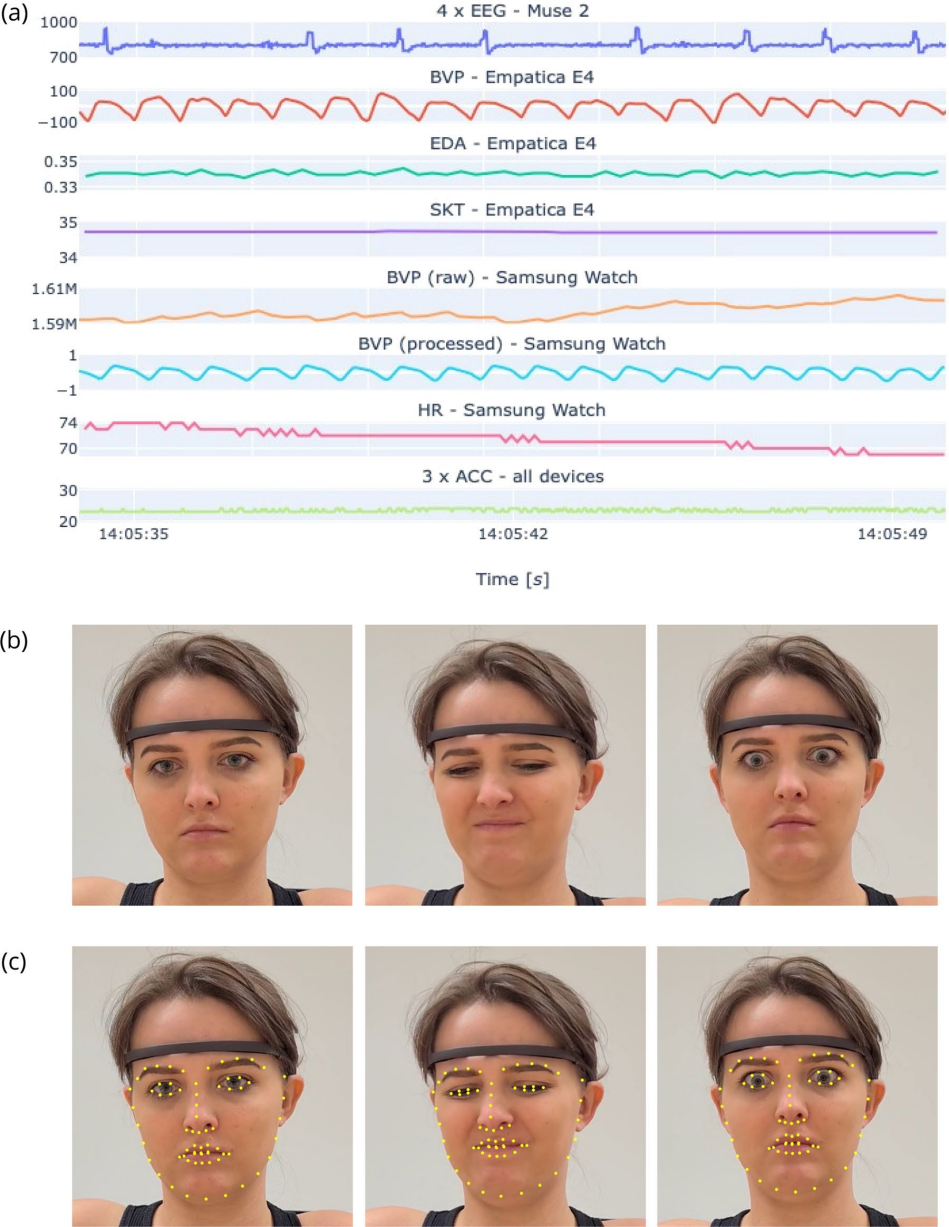
Examples of data available in the Emognition dataset: (**a**) physiological signals recorded with wearable devices: 4 x EEG (Muse 2); BVP, EDA, SKT (Empatica E4); raw BVP, processed BVP, HR (Samsung Watch); ACC from all devices; (**b**) upper-body recordings capturing the facial reactions to the stimuli, from the left: neutral, disgust, surprise; (**c**) facial landmarks generated with the *OpenFace* library facilitating emotion recognition from the face. The participant gave written consent to include her image in this article.

Collected data (physiological signals, upper-body recordings, self-reports, and control questionnaires) are available at Harvard Dataverse Repository^45^. The types of data available in the Emognition dataset are illustrated in Fig. 5. The upper-body recordings in an MP4 format, full HD resolution (1920 × 1080) constitute 76GB of space. The other data is compressed into the *study_data.zip* package of size 1GB (16GB after decompression). The data are grouped by participants. Each participant has their folder containing files from all experimental stages (stimulus presentation, washout, self-assessment) and all devices (Muse 2, Empatica E4, Samsung Watch). In total, each participant has 97 files related to:

- 10 film clips × 3 devices × 3 phases (washout, stimulus, self-assessment) = 90 files with signals;
- baseline × 3 devices × 2 phases (baseline, self-assessment) = 6 files with signals;
- a *questionnaires.json* file containing self-assessment responses, the control questionnaire, and some metadata (demographics and information about wearing glasses, e.g.).

Additionally, facial annotations are provided in two ZIP packages, *OpenFace.zip* and *Quantum.zip*, respectively. The *OpenFace* package contains facial landmark points and action units’ values (7.4GB compressed, 25GB after decompression). The *Quantum Sense* package contains values of six basic emotions and head position (0.7GB compressed, 4.7GB after decompression). The values are assigned per video frame.

The files are in JSON format, except *OpenFace* annotations in CSV format. More technical information (e.g., file naming conventions, variables available in each file) is provided in the *README.txt* file included in the dataset.

## Technical Validation

To test whether film clips elicit targeted emotions, we used repeated-measures analysis of variance (rmANOVA) with Greenhouse-Geisser correction and calculated recommended effect sizes of ${\eta }_{p}^{2}$ for ANOVA tests^46,47^. To examine differences between the conditions (e.g., whether self-reported amusement in response to the amusing film clips was higher than it was reported in response to the other film clips), we calculated pairwise comparisons with Bonferroni correction of *p*-values for multiple comparisons.

**Fig. 6:**
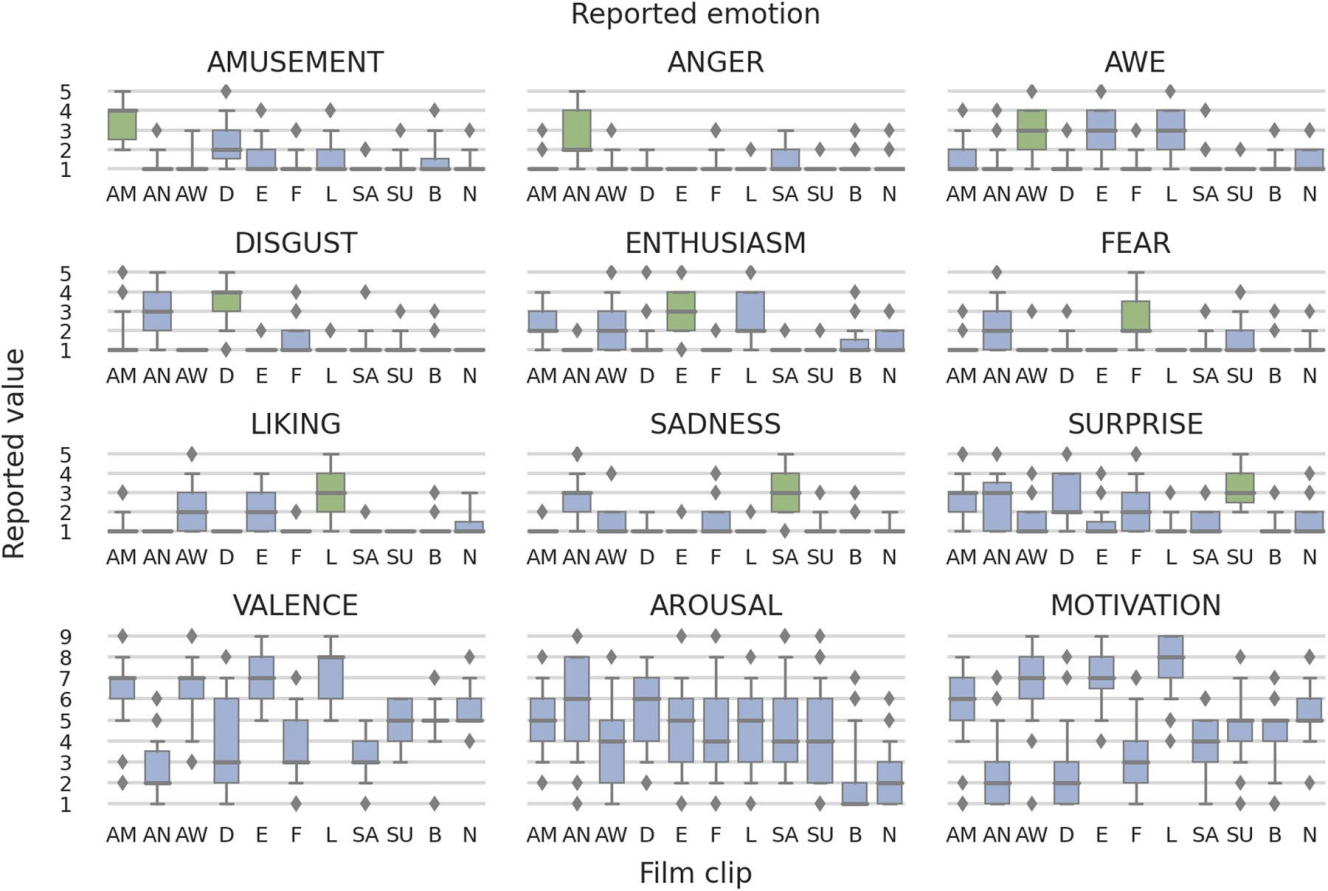
Distribution of self-reported emotions between conditions, i.e., what level of a given emotion was elicited by different films (conditions). The chart titles indicate reported emotions, vertical axis denotes the values used in questionnaires (intensity of emotions: 1–5 for discrete emotions, 1–9 for SAM), and X-axis labels represent film clips (conditions); AM - amusement, AN - anger, AW - awe, D - disgust, E - enthusiasm, F - fear, L - liking, SA - sadness, SU - surprise, B - baseline, N - neutral. Green indicates targeted emotion. Boxes depict quartiles of distributions and whiskers the span from the 5th to 95th percentile. Everything outside them (diamonds) is classified as outliers.

**Fig. 7:**
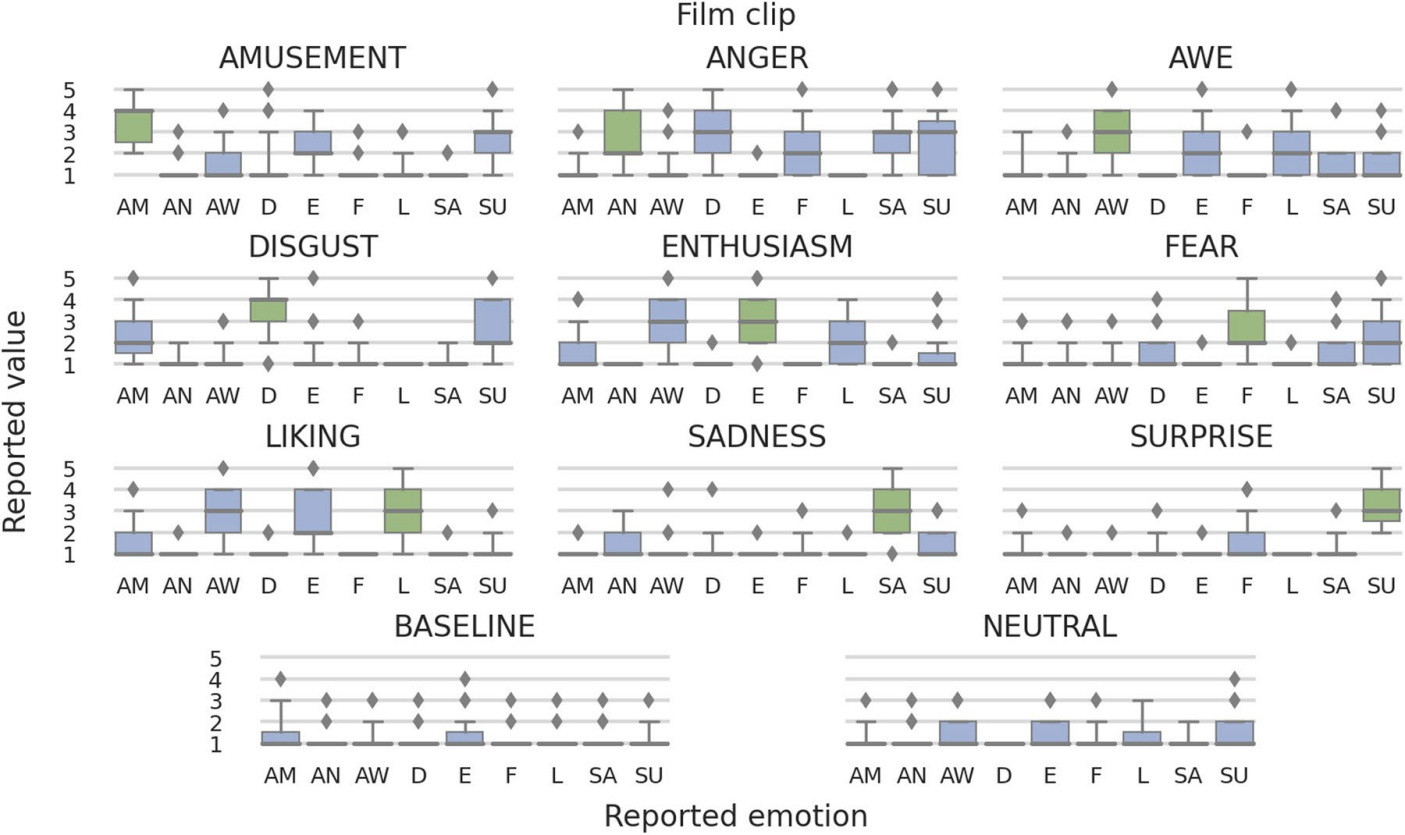
Distribution of self-reported discrete emotions within conditions, i.e., what really emotions were evoked by films that were supposed to invoke a given emotion. The chart titles denote film clips (conditions). Vertical axis corresponds to the values used in the questionnaire (intensity of emotions). The horizontal labels represents discrete emotions reported by the participants: AM - amusement, AN - anger, AW - awe, D - disgust, E - enthusiasm, F - fear, L - liking, SA - sadness, SU - surprise. Boxes present quartiles of distributions, whereas whiskers – the span from the 5th to 95th percentile. Everything outside them is treated as outliers (diamonds in the graph).

As summarized in Tables 3, 4, Figs. 6 and 7, watching film clips evoked the targeted emotions. The differences in self-reported emotions in film clips should be interpreted as large^48^. Pairwise comparisons indicated that self-reported targeted emotions were the highest in the corresponding film clip condition (e.g., self-reported amusement in response to the amusing film clip). Furthermore, we observed that some emotions were intense in more than one film clip condition and some film clips elicited more than one emotion. These are frequent effects during emotion elicitation procedures^22,29,30^, see Supp. Mat. for details.

**Table 3:** Results of Repeated Measures Analysis of Variance for Differences Between Conditions in Self-reported Emotions.

Self-reports	Film Clips																						rm ANOVA		
	Amusement		Anger		Awe		Disgust		Enthusiasm		Fear		Liking		Sadness		Surprise		Baseline		Neutral				
	M	SD	M	SD	M	SD	M	SD	M	SD	M	SD	M	SD	M	SD	M	SD	M	SD	M	SD	F	df	${{\boldsymbol{\eta }}}_{{\boldsymbol{p}}}^{{\boldsymbol{2}}}$
Amusement	3.37	0.98	1.16	0.43	1.3	0.64	2.33	1.11	1.47	0.85	1.3	0.6	1.6	0.85	1.07	0.26	1.26	0.49	1.37	0.72	1.14	0.41	45.37***	5.32, 223.57	0.52
Anger	1.09	0.37	2.7	1.26	1.14	0.41	1.19	0.39	1	0	1.28	0.59	1.02	0.15	1.47	0.67	1.02	0.15	1.07	0.34	1.09	0.37	38.21***	2.96, 124.23	0.48
Awe	1.42	0.73	1.16	0.57	2.86	1.08	1.14	0.47	2.74	1.18	1.12	0.39	2.74	1.07	1.12	0.5	1.02	0.15	1.19	0.5	1.4	0.62	49.04***	4.80, 201.76	0.54
Disgust	1.4	0.93	2.84	1.25	1	0	3.49	1.14	1.02	0.15	1.47	0.7	1.05	0.21	1.26	0.58	1.14	0.41	1.07	0.34	1	0	70.17***	3.97, 166.75	0.63
Enthusiasm	2.3	0.99	1.02	0.15	2.37	1.2	1.35	0.78	2.95	1.05	1.02	0.15	2.58	1.22	1.02	0.15	1.02	0.15	1.35	0.69	1.35	0.61	46.60***	4.06, 170.42	0.53
Fear	1.07	0.34	2.19	1.07	1.05	0.3	1.26	0.49	1	0	2.7	1.17	1	0	1.26	0.54	1.6	0.9	1.07	0.34	1.21	0.47	37.57***	4.09, 171.88	0.47
Liking	1.26	0.58	1	0	2.44	1.22	1	0	2.14	1.1	1.05	0.21	3.16	1.19	1.02	0.15	1	0	1.07	0.34	1.35	0.65	56.76***	3.64, 152.92	0.57
Sadness	1.05	0.21	2.65	1.11	1.42	0.73	1.19	0.39	1.02	0.15	1.44	0.77	1.02	0.15	3.16	1.02	1.12	0.39	1.09	0.37	1.23	0.43	68.50***	4.30, 180.45	0.62
Surprise	2.58	1.1	2.49	1.24	1.42	0.7	2.56	1.2	1.33	0.64	2.14	1.19	1.26	0.49	1.42	0.63	3.33	1.02	1.21	0.47	1.51	0.7	36.40***	6.64, 279.08	0.46
Valence	6.58	1.43	2.6	1.35	6.47	1.42	3.91	2.09	6.98	1.24	3.65	1.54	7.3	1.28	3.37	0.9	4.86	1.04	5.02	0.94	5.35	0.78	64.25***	5.31, 222.87	0.6
Arousal	4.95	1.54	5.79	2.16	4.05	2.03	5.33	1.82	4.56	1.99	4.51	2.13	4.6	1.93	4.44	2.07	4.07	2.11	1.98	1.55	2.37	1.33	25.57***	6.50, 272.93	0.38
Motivation	5.84	1.65	2.65	1.6	7.07	1.56	2.4	1.72	7.23	1.39	3.02	1.67	7.56	1.28	3.6	1.43	4.79	1.34	4.42	1.22	5.51	1.18	78.69***	6.99, 293.55	0.65

*Note*. The significant results of repeated measures analysis of variance indicates differences in self-reported emotions between film clip conditions (e.g., differences in self-reported amusement between amusing film clip and sad film clip, angry film clip etc.). M = Mean, SD = Standard Deviation, F = F-Ratio calculated by dividing the mean squares for the variable by its error mean squares, ***p 0.001.

**Table 4:** Results of Repeated Measures Analysis of Variance for Differences Within Conditions in Self-reported Emotions.

Film Clips	Self-reports																		rm ANOVA		
	Amusement		Anger		Awe		Disgust		Enthusiasm		Fear		Liking		Sadness		Surprise				
	M	SD	M	SD	M	SD	M	SD	M	SD	M	SD	M	SD	M	SD	M	SD	F	df	${{\boldsymbol{\eta }}}_{{\boldsymbol{p}}}^{{\boldsymbol{2}}}$
Amusement	3.37	0.98	1.09	0.37	1.42	0.73	1.4	0.93	2.3	0.99	1.07	0.34	1.26	0.58	1.05	0.21	2.58	1.1	57.63***	3.69, 154.91	0.58
Anger	1.16	0.43	2.7	1.26	1.16	0.57	2.84	1.25	1.02	0.15	2.19	1.07	1	0	2.65	1.11	2.49	1.24	38.26***	4.52, 189.84	0.48
Awe	1.3	0.64	1.14	0.41	2.86	1.08	1	0	2.37	1.2	1.05	0.3	2.44	1.22	1.42	0.73	1.42	0.7	39.04***	3.62, 152.09	0.48
Disgust	2.33	1.11	1.19	0.39	1.14	0.47	3.49	1.14	1.35	0.78	1.26	0.49	1	0	1.19	0.39	2.56	1.2	58.73****	3.50, 146.79	0.58
Enthusiasm	1.47	0.85	1	0	2.74	1.18	1.02	0.15	2.95	1.05	1	0	2.14	1.1	1.02	0.15	1.33	0.64	64.86***	3.08, 129.23	0.61
Fear	1.3	0.6	1.28	0.59	1.12	0.39	1.47	0.7	1.02	0.15	2.7	1.17	1.05	0.21	1.44	0.77	2.14	1.19	29.08***	3.50, 146.95	0.41
Liking	1.6	0.85	1.02	0.15	2.74	1.07	1.05	0.21	2.58	1.22	1	0	3.16	1.19	1.02	0.15	1.26	0.49	71.87***	2.64, 110,79	0.63
Sadness	1.07	0.26	1.47	0.67	1.12	0.5	1.26	0.58	1.02	0.15	1.26	0.54	1.02	0.15	3.16	1.02	1.42	0.63	66.77***	3.83, 160.79	0.62
Surprise	1.26	0.49	1.02	0.15	1.02	0.15	1.14	0.41	1.02	0.15	1.6	0.9	1	0	1.12	0.39	3.33	1.02	94.21***	2.59, 108.926	0.69
Baseline	1.37	0.72	1.07	0.34	1.19	0.5	1.07	0.34	1.35	0.69	1.07	0.34	1.07	0.34	1.09	0.37	1.21	0.47	5.33***	2.53, 106.07	0.11
Neutral	1.14	0.41	1.09	0.37	1.4	0.62	1	0	1.35	0.61	1.21	0.47	1.35	0.65	1.23	0.43	1.51	0.7	5.34***	4.99, 209.62	0.11

*Note*. The significant results of repeated measures analysis of variance indicates differences in self-reported emotions within film clip conditions (e.g., differences in amusing film clip between self-reported amusement and sadness, anger etc.). M = Mean, SD = Standard Deviation, F = F-Ratio calculated by dividing the mean squares for the variable by its error mean squares, ***p 0.001.

To validate the quality of the recorded physiological signals, we computed signal-to-noise ratios (SNRs) by fitting the second-order polynomial to the data obtained from the autocorrelation function. It was done separately for all physiological recordings (all participants, baselines, film clips, and experimental stages, see Sec. Data Records). SNR statistics indicated the signals’ high quality. Mean SNR ranged from 26.66 dB to 37.74 dB, with standard deviations from 2.27 dB to 11.13 dB. For one signal, the minimum SNR was 0.88 dB. However, 99.7% of its recordings had SNR values over 5.15 dB. As the experiments were conducted in a sitting position, we did not analyze signals from accelerometers and gyroscopes. For details, see Supp. Mat. Table 3.

Additionally, the *Quantum Sense* annotations were analyzed to see how well the software recognized emotions. In general, it performed well within conditions, but poorly between conditions. The main reason behind wrong or missing annotations were participants covering face with palm or leaning towards camera. In some cases, participants already seen the movie and react differently – smiled instead of being disguised. For details see Supp. Mat. Sec. *Analysis of Quantum Sense Results*.

## Usage Notes

### Emotion Recognition

The most common approach to emotion recognition from physiological signals includes (1) data collection and cleaning; (2) signal preprocessing, synchronization, and integration; (3) feature extraction and selection; and (4) machine learning model training and validation. A comprehensive overview of all these stages can be found in our review on emotion recognition using wearables^49^.

For further processing of the Emognition dataset, we recommend the following Python libraries, which we analyzed and found useful for feature extraction from physiological data. The *pyPhysio* library^50^ (https://github.com/MPBA/pyphysio) facilitates the analysis of ECG, BVP, and EDA signals by providing algorithms for filtering, segmentation, extracting derivatives, and other signal processing. The *BioSPPy* library (https://biosppy.readthedocs.io/) handles BVP, ECG, EDA, EEG, EMG, and respiration signals. For example, it filters BVP, performs R-peak detection, and computes the instantaneous HR. The *Ledalab* library^51^ focuses on the EDA signal and offers both continuous and discrete decomposition analyses. The *Kubios* software (https://www.kubios.com) enables data import from several HR, ECG, and PPG monitors, and calculates over 40 features from the HRV signal. The *PyEEG* library (https://github.com/forrestbao/pyeeg) is intended for EEG signal analysis and processing, but it works with any time series data.

Emotion recognition from facial expressions can be achieved by processing video images^52^. At first, the face has to be detected, and landmarks (distinctive points in facial regions) need to be identified. Tracing the position of landmarks between video frames allows us to measure muscle activity and encode it into Action Units (AUs). AUs can be used to identify emotions as proposed by Ekman and Friesen in their Facial Action Coding System (FACS)^53^.

### Accessing data

The use of the Emognition dataset is limited to academic research purposes only due to the consent given by the participants. The data will be made available after completing the End User License Agreement (EULA). The EULA is located in the dataset repository. It should be signed and emailed to the Emognition Group at mailto:emotions@pwr.edu.pl. The mail has to be sent from an academic email address associated with the Harvard Dataverse platform account.

## Supplementary information

Supplementary Materials

## Data Availability

The code used for the technical validation is publicly available at https://github.com/Emognition/Emognition-wearable-dataset-2020. The code was developed in Python 3.7. The repository contains several *Jupyter Notebooks* with data manipulations and visualizations. All required packages are listed in *requirements.txt* file. The repository may be used as a starting point for further data analyses. It allows you to easily load and preview the Emognition dataset.
