# Supplementary material for: Emognition dataset: emotion recognition with self-reports, facial expressions, and physiology using wearables
Source: Sci Data. 2022 Apr 7;9:158. doi: 10.1038/s41597-022-01262-0 (PMC8989970; doi:10.1038/s41597-022-01262-0)
Supplement: Supplementary file 1 — Supplementary Materials [file 41597_2022_1262_MOESM1_ESM.pdf]

Emognition dataset: emotion recognition with self-reports, facial expressions, and physiology using wearables – Supplementary Materials

Stanisław Saganowski [1,*], Joanna Komoszyńska [1], Maciej Behnke [1,2], Bartosz Perz [1], Dominika Kunc [1], Bartłomiej Klich [1], Łukasz D. Kaczmarek [2], and Przemysław Kazienko [1]

[1]Wroclaw University of Science and Technology, Faculty of Information and Communication Technology, Department of Artificial Intelligence, Wrocław, 50-370, Poland
[2]Adam Mickiewicz University, Faculty of Psychology and Cognitive Science, Poznan, 61-664, Poland
[*]corresponding author: Stanisław Saganowski (stanislaw.saganowski@pwr.edu.pl)

Detailed Results of Self-reports

In this section, we provide a detailed description of the obtained p-values. Tab. 1 contains p-values between emotion in the respective condition and the same emotion in other conditions, e.g. disgust for the film expected to elicit disgust (the same condition) was significantly different from disgust reported for the awe film – another condition ($p < 0.001$), however, it was not different from disgust in the angry film condition ($p = 0.364$). Tab. 2 contains p-values between emotion expected in respective condition and other emotions within the same condition, e.g. anger in the angry film condition was significantly different from enthusiasm in the angry film condition ($p < 0.001$), but it was not different from disgust in angry film condition ($p = 1.0$).

Difference Between Film Clips. Pairwise comparisons, obtained with the one-way repeated measures ANOVA, indicated that self-reported targeted emotions were the highest in the corresponding film clip condition, all *p*s < .001 (Tab. 1). It means that amusement was higher in the amusing film condition than in other film conditions, all p-values (*p*s) < .001. Similarly, anger was higher in the angry film condition than in other film conditions, all *p*s < .001, etc.

However, in other films clips conditions, some emotions were taking high values in more than one film clip condition. Awe was higher in the awe film condition than in other film conditions, all *p*s < .001, except for liking and enthusiastic conditions *p*s = 1.00. Disgust was higher in the disgusting film condition than in other film conditions, all *p*s < .001, except for angry film condition $p = .36$. Enthusiasm was higher in the enthusiastic film condition than in other film conditions, all *p*s < .05, except for liking film condition $p = 1.00$. Fear was higher in the fearful film condition than in other film conditions, all *p*s < .001, except for angry film condition $p = .49$. Liking was higher in the liking film condition than in other film conditions, all *p*s < .01. Sadness was higher in the sad film condition than in other film conditions, all *p*s < .001. except for angry film condition p = .25. Surprise was higher in the surprising film condition than in other film conditions, all *p*s < .05, except for disgust film condition $p = .06$.

Furthermore, we found that the liking, enthusiastic, amusing, and awe film clips were more positively valenced than baseline and neutral film clips all *p*s < .001, indicating the group of positive emotions. The angry, disgusting, fearful, and sad movies were more negatively valenced than baseline and neutral film clips, all *p*s < .05, indicating the group of negative emotions. In terms of valence, surprise did not differ from the baseline and neutral film clips, both *p*s = 1.00. Affective clips were arousing relative to baseline levels and to the neutral film clips, all *p*s < .001. Neutral film clip elicited stronger approach motivation than baseline. Liking, enthusiastic, and awe clips elicited stronger approach motivation than neutral film clip, all *p*s < .001, indicating the group of approach motivated emotions. The film clips for anger, disgust, fear, and sadness elicited stronger avoidance motivation indicating the group of avoidance motivated emotions. Amusement and surprise did not differ from the neutral film clip in arousal, both *p*s > .05.

Difference Within Film Clips. Self-reported targeted emotions were highest in the corresponding film clip condition (Tab. 2). In amusement film condition, self-reported amusement was higher than self-reported anger, awe, disgust, enthusiasm, fear, liking, sadness, and surprise, all *p*s < .001. Similarly, disgust was higher than other self-reported emotions in disgust film condition, all *p*s < .002; sadness was higher than other self-reported emotions in sadness film condition, all *p*s < .001; and surprise was higher than other self-reported emotions in surprise film conditions, all *p*s < .001. In some cases, however, more than one emotion took high values within the particular stimulus. In the angry film clip condition, anger was higher than other self-reported positive emotions (amusement, awe, enthusiasm, liking), all *p*s < .001, but did not differ from other negative emotions (disgust, fear, and sadness), and surprise, all *p*s > .05. Moreover, distributions of anger and disgust were very similar,

with the median higher in the latter emotion. Awe was higher than other self-reported emotions in awe film condition, all $ps < .01$, except for self-reported liking, $p = .90$. Enthusiasm was higher than other self-reported emotions in the enthusiasm film condition, all $ps < .001$, except for self-reported awe, $p = .99$. Fear was higher than other self-reported emotions in the fear film condition, all $ps < .001$, except for self-reported surprise, $p = .05$. Liking was higher than other self-reported emotions in the liking film condition, all $ps < .003$, except for self-reported awe, $p = .05$. There were no differences between self-reported emotions after watching the baseline film , all $ps > .05$.

Self-report	Film clip Amusement	Anger	Awe	Disgust	Enthusiasm	Fear	Liking	Sadness	Surprise	Baseline	Neutral
Amusement		< 0.001	< 0.001	< 0.001	< 0.001	< 0.001	< 0.001	< 0.001	< 0.001	< 0.001	< 0.001
Anger	< 0.001		< 0.001	< 0.001	< 0.001	< 0.001	< 0.001	< 0.001	< 0.001	< 0.001	< 0.001
Awe	< 0.001	< 0.001		< 0.001	1.0	< 0.001	1.0	< 0.001	< 0.001	< 0.001	< 0.001
Disgust	< 0.001	0.364	< 0.001		< 0.001	< 0.001	< 0.001	< 0.001	< 0.001	< 0.001	< 0.001
Enthusiasm	0.006	< 0.001	0.026	< 0.001		< 0.001	1.0	< 0.001	< 0.001	< 0.001	< 0.001
Fear	< 0.001	0.488	< 0.001	< 0.001	< 0.001		< 0.001	< 0.001	< 0.001	< 0.001	< 0.001
Liking	< 0.001	< 0.001	0.017	< 0.001	0.003	< 0.001		< 0.001	< 0.001	< 0.001	< 0.001
Sadness	< 0.001	0.253	< 0.001	< 0.001	< 0.001	< 0.001	< 0.001		< 0.001	< 0.001	< 0.001
Surprise	0.016	0.006	< 0.001	0.064	< 0.001	< 0.001	< 0.001	< 0.001		< 0.001	< 0.001

Table 1. *P*-values from repeated measures analysis of variance (rANOVA) for differences between conditions (different films – columns) in self-reported emotions

Film clip	Self-report Amusement	Anger	Awe	Disgust	Enthusiasm	Fear	Liking	Sadness	Surprise
Amusement		< 0.001	< 0.001	< 0.001	< 0.001	< 0.001	< 0.001	< 0.001	0.001
Anger	< 0.001		< 0.001	1.0	< 0.001	0.479	< 0.001	1.0	1.0
Awe	< 0.001	< 0.001		< 0.001	0.013	< 0.001	0.896	< 0.001	< 0.001
Disgust	0.002	< 0.001	< 0.001		< 0.001	< 0.001	< 0.001	< 0.001	< 0.001
Enthusiasm	< 0.001	< 0.001	1.0	< 0.001		< 0.001	< 0.001	< 0.001	< 0.001
Fear	< 0.001	< 0.001	< 0.001	< 0.001	< 0.001		< 0.001	< 0.001	0.053
Liking	< 0.001	< 0.001	0.117	< 0.001	0.003	< 0.001		< 0.001	< 0.001
Sadness	< 0.001	< 0.001	< 0.001	< 0.001	< 0.001	< 0.001	< 0.001		< 0.001
Surprise	< 0.001	< 0.001	< 0.001	< 0.001	< 0.001	< 0.001	< 0.001	< 0.001	

Table 2. *P*-values from repeated measures analysis of variance (rANOVA) for differences within conditions (for the film being supposed to elicit a given emotion) in self-reported emotions

	MUSE TP9	AF7	AF8	TP10	Samsung BVP	Empatica BVP	TEMP	EDA
count	1312	1312	1312	1312	1344	1350	1348	1350
mean	36.22	37.74	37.12	37.16	33.77	33.43	26.81	26.66
std	6.02	9.44	11.13	6.77	2.86	2.27	3.04	2.81
min	0.88	5.85	4.13	4.84	17.76	23.57	3.01	18.50
Q0.3	5.15	8.74	7.26	7.39	26.24	25.51	17.48	19.78
Q25	33.74	37.50	38.18	35.31	31.40	32.12	24.46	24.36
Q50	37.46	40.84	41.39	39.12	35.46	33.54	28.56	27.90
Q75	40.17	43.47	43.99	41.12	35.82	34.93	28.84	28.68
max	45.63	48.62	49.88	46.73	42.91	40.83	36.91	37.00

Table 3. Statistics for signal-to-noise ratios (SNRs) computed for raw physiological signals recorded during the study. Columns inherit their names after abbreviations of signals (BVP – blood volume pulse; TEMP – skin temperature; EDA – electrodermal activity). All values are in decibels (dB), except for count. For the MUSE device, all column names correspond to locations of the electrodes used for recording electroencephalograms (EEGs). Qx are values for quantiles, where x denotes the percentage of samples falling into the bin from min to Qx, e.g., 0.3 % of computed SNRs have values below Q0.3, and Q50 is the median. Differences in samples used for calculations (count) come from the malfunctioning of the devices, see Sec. *Data Processing and Cleaning* in the Main File.

Analysis of obtained signal-to-noise ratios (SNRs)

A detailed analysis of the obtained signal-to-noise ratios (SNRs) is provided in this section.

We chose to analyze only raw signals provided by the devices, described in Main File Sec. *Data Processing and Cleaning*. Moreover, we did not analyze signals from accelerometers and gyroscopes, as the experiments were conducted in a sitting position. Implementation of the algorithm used to calculate SNR is provided in the Main File Sec. *Technical Validation*. Tab. 3 contains detailed information regarding statistics of SNR values obtained for particular signals. We discarded samples where the sensors malfunctioned, thus, some differences in samples taken into consideration (count) are present.

For electroencephalography (EEG) signals, the mean SNR values were between 36 dB and 38 dB with standard deviations (std) from 6.02 dB to 11.13 dB. Medians were slightly higher: from 37.46 dB to 41.39 dB. The lowest SNR obtained was 0.88 dB (for TP9 electrode). However, only 0.3% of recordings from this electrode had values below 5.15 dB. For other EEG electrodes, the computed SNR was never below 4.13 dB.

Blood volume pulse (BVP) collected from Samsung smartwatch had the mean SNR of 33.77 dB with std of 2.86 dB, and the median SNR of 35.46 dB. Moreover, the minimum value of the computed ratio was 17.76 dB, and only 0.3% of samples had SNR values below 26.24 dB.

SNRs computed for the signals acquired by means of the Empatica device reached the average values from 26.66 dB for electrodermal activity (EDA) to 33.43 dB for BVP, with standard deviations from 2.27 for BVP to 3.04 for temperature (TEMP). The lowest SNR=3.01 dB was received for TEMP. Nevertheless, the temperature SNRs were below 17.48 dB only for 0.3% of recorded cases. For other signals from Empatica, SNRs were never below 18.5 dB for EDA and 23.57 dB for BVP.

Analysis of Quantum Sense Results

We have analyzed the Quantum Sense results in relation to the targeted emotion (stimuli annotation). Please note that such analysis validates the Quantum Sense annotations only, not the study procedure nor stimuli effectiveness. The latter is validated in the Main File Sec. *Technical Validation*.

The videos were processed using Quantum Sense software to perform emotion classification with six basic emotions (neutral, anger, disgust, happiness, sadness, surprise). The software classified each video frame if a human face was detected. The annotated frames from each participant were grouped by the stimuli type (within the condition) or by the annotations from the software (between conditions). Then, the number of frames was changed to the percentage of frames classified with a specific emotion within a given stimulus. We noticed that participants had a neutral face over the majority of the experiment. They expressed emotions very briefly when stimuli had more intense/key moments. Hence, most of the frames (87% of all recordings) were labeled with neutral state. The distribution of expressed emotions averaged over all participants within and between conditions is presented in Figs. 1 and 2, respectively. The neutral state is not presented because it would make other bars barely visible.

The Quantum Sense software clearly detected the targeted emotion in amusement and sadness stimuli. Less obvious but

still in line with the targeted emotion are annotations in enthusiasm, liking, and anger stimuli. In disgust, fear, and baseline stimuli, the detected expressions relate mainly to happiness, while sadness dominates in awe, neutral, and surprise stimuli.

For some stimuli, the results are not as expected. This, however, may be caused by strong, not always true, assumptions, i.e., (1) as ground truth, we assumed the stimuli annotation instead of the participants' response; (2) we assume that participants facially express the targeted emotion. Some clips were known to the participants and could have invoked different emotions/expressions than targeted, e.g., disgust stimulus was funny to people who had already seen the movie. Moreover, the Quantum Sense software does not perform the neutral face calibration. Some people have naturally down-turned corners of the mouth, which the software can classify as sadness. Further, if a person skipped a film clip, there were fewer frames with an emotion, or the person could not reach a key moment of the stimuli.

We encourage readers to explore this topic further using OpenFace annotations or self-developed landmark detection algorithms.

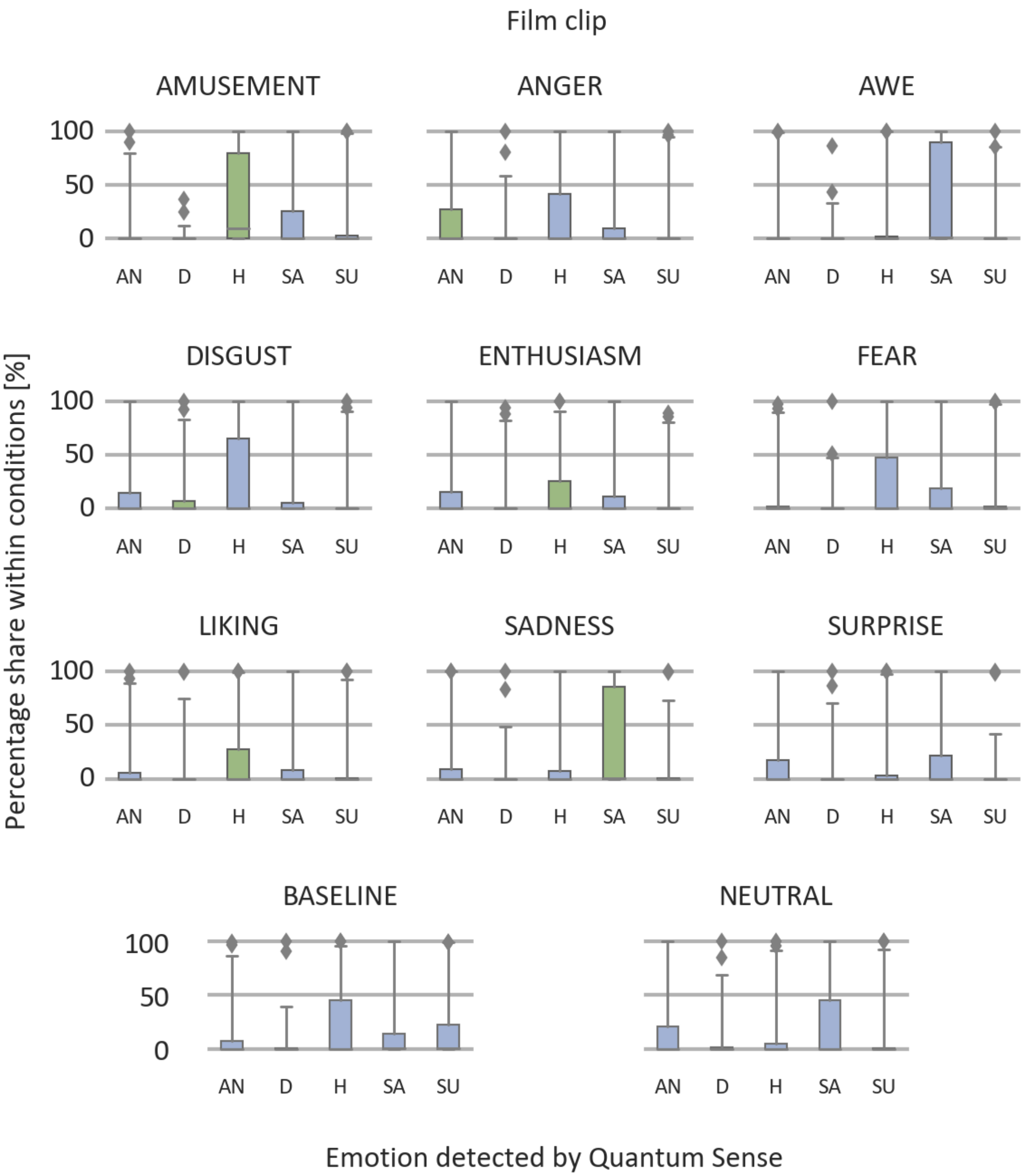

Figure 1. Percentage share of emotions detected by Quantum Sense (Research Edition 2017, Quantum CX, Poland) within conditions, i.e., for a given film reflected by the chart title. Vertical scales denote the percentage of frames classified with the emotion labeled horizontally to all frames with detected any emotion for the film from the title. These values are grouped for each participant. The horizontal detected discrete emotions are: AN - anger, D - disgust, H - happiness, SA - sadness, SU - surprise. Boxes depict quartiles of distributions, whiskers – the span from the 5th to 95th percentile, diamonds – all other values (outliers).

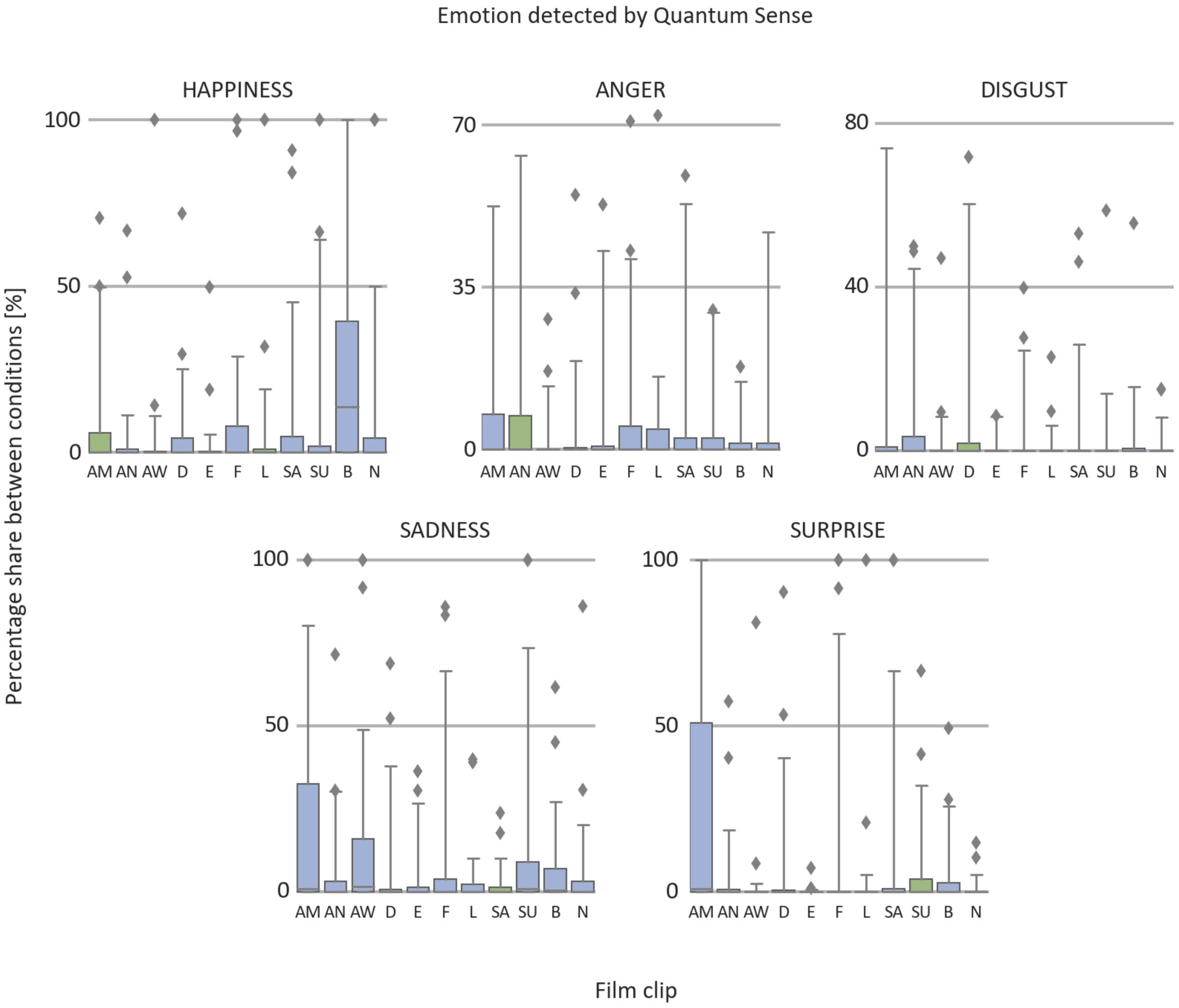

Figure 2. Percentage share of emotions detected by Quantum Sense (Research Edition 2017, Quantum CX, Poland) between conditions, i.e., how much a given emotion was evoked by different films (conditions). Chart titles correspond to emotions detected by the software. Y-axis values represents the percentage of frames classified with the same emotion as the title among all frames with any detected emotion for the film labelled horizontally. These values are grouped for each participant. The film clips (conditions) are: AM - amusement, AN - anger, AW - awe, D - disgust, E - enthusiasm, F - fear, L - liking, SA - sadness, SU - surprise, B - baseline, N - neutral. Green objects indicate the targeted emotion. Boxes represent quartiles of distributions, while whiskers – the span from the 5th to 95th percentile. Diamonds are outliers out of the whiskers.

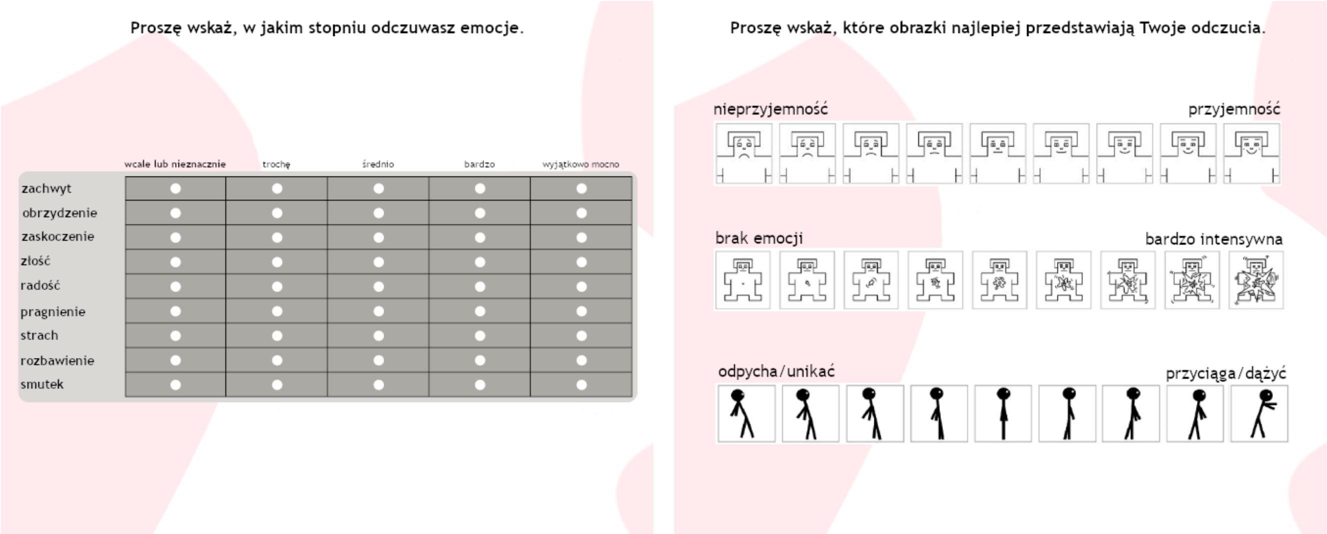

(a) Questionnaire for discrete emotions.

(b) Questionnaire for valence, arousal, and motivation.

Figure 3. The original (Polish) version of self-reports used in the study.
